# Labeling Microplastics with Fluorescent Dyes for Detection, Recovery, and Degradation Experiments

**DOI:** 10.3390/molecules27217415

**Published:** 2022-11-01

**Authors:** Zhiqiang Gao, Kendall Wontor, James V. Cizdziel

**Affiliations:** Department of Chemistry and Biochemistry, University of Mississippi, University, MS 38677, USA

**Keywords:** microplastics, fluorescence, textile dyes, Nile red, hydrophobicity

## Abstract

Staining microplastics (MPs) for fluorescence detection has been widely applied in MP analyses. However, there is a lack of standardized staining procedures and conditions, with different researchers using different dye concentrations, solvents, incubation times, and staining temperatures. Moreover, with the limited types and morphologies of commercially available MPs, a simple and optimized approach to making fluorescent MPs is needed. In this study, 4 different textile dyes, along with Nile red dye for comparison, are used to stain 17 different polymers under various conditions to optimize the staining procedure. The MPs included both virgin and naturally weathered polymers with different sizes and shapes (e.g., fragments, fibers, foams, pellets, beads). We show that the strongest fluorescence intensity occurred with aqueous staining at 70 °C for 3 h with a dye concentration of 5 mg/mL, 55 mg/mL, and 2 µg/mL for iDye dyes, Rit dyes, and Nile red, respectively. Red fluorescent signals are stronger and thus preferred over green ones. The staining procedure did not significantly alter the surface, mass, and chemical characteristics of the particles, based on FTIR and stereomicroscopy. Stained MPs were spiked into freshwater, saltwater, a sediment slurry, and wastewater-activated sludge; even after several days, the recovered particles are still strongly fluoresced. The approach described herein for producing customized fluorescent MPs and quantifying MPs in laboratory-controlled experiments is both straightforward and simple.

## 1. Introduction

Microplastics (MPs) have been found nearly everywhere in the environment, including in surface water, both freshwater [1,2] and seawater [3], the atmosphere [4], sand [5], organisms [6], and foodstuffs [7,8]. This overwhelming prevalence is concerning because MPs are potentially harmful in several aspects. Not only can they potentially cause physical damage to organs, but they may also be toxic due to both their chemical or elemental additives as well as the pollutants they adsorb from the environment [9]. Many studies have therefore investigated the occurrence, fate, and identification of MPs due to their pervasiveness and potential for harm. However, the absence of an established systemically harmonized MP analysis procedure makes it challenging to compare results within these studies. This is further compounded by the lack of commercially available MPs that closely resemble weathered (environmentally aged) MPs, although some metrology institutes such as BAM in Germany now offer MPs that are “artificially aged” with UV light. Without environmentally relevant certified standards, it becomes difficult to not only compare the results of various studies but also to assess recoveries from sampling and analysis procedures.

It is common to use fluorescent MPs, such as polystyrene (PS) and polyethylene (PE) microbeads, to study MPs in laboratory experiments [10,11,12]. For instance, 20 μm commercial fluorescent PS microbeads were used to examine their adsorption onto edible seaweed and their ability to be washed off [13]. Smaller plastic microbeads (1–5 μm) were used to characterize MP ingestion and their effects on the behaviors of oyster larvae [14]. While convenient due to commercial availability, microbeads are not representative of environmental MPs, as most MPs extracted from water, sediments, atmosphere, and food are typically weathered fibers, fragments, or films. Further, microbeads are less common in the natural environment and are increasingly being banned in personal care products [15,16,17]. Additionally, although PE and PS account for 36% of global plastic production, other types of polymers, such as PVC (10%), polyurethane (7.9%), and PC (1.5%), are considered more hazardous based on their monomer composition and additives [18]. This suggests that MP toxicity studies based primarily on PE and PS microbeads may be heavily biased.

In addition to microbeads, MP fragments (>500 µm) have been used to assess methodology due to their commercial availability and easy identification [19,20,21]. However, this size category generally accounts for a small proportion of MPs extracted from environmental and biological samples. The larger-sized MPs may also not respond to sample preparation methods in the same way as the more abundant smaller MPs. As such, their recovery rates should not be used to correct MP abundances in environmental samples [22].

Due to the lack of appropriate standards, a variety of dyes have been proposed to fluorescently label homemade plastic particles, enriching available MP morphologies and polymer types. These dyes include Nile red and its derivatives, oil red EGN, Rose Bengal, Neutral Red, Tryphan Blue, and some commercial textile dyes [12,23,24,25,26]. While all these dyes have proven suitable for staining MPs, each dye’s affinity for different polymers varies. Nile red is the most used dye for MP staining. However, it is poorly soluble in water and exhibits a significant chromophore sensitivity to solvent polarity (Martinez and Henary, 2016). Due to this, the color emission of MPs stained by Nile red depends on MP surface hydrophobicity and can range from deep red to strong yellow gold. One concern with the use of Nile Red is that MPs hydrophobicity could be altered due to contamination from other materials (e.g., biofilms, grease). To avoid this issue, several commercial textile dyes containing aromatic amines (iDye pink, iDye blue, Rit pink, and Rit blue) have also been studied. These textile dyes performed well for PS, PET, and PVC fragments, HDPE microspheres, PET, and PAN fibers with signals that were several times stronger than that of Nile Red in most cases [12].

While a variety of dyes have been investigated for use in MP staining, the scientific literature lacks an overall standardized method for staining MPs. Indeed, even among studies using the same dye, there is a diversity of methods, with each using different conditions such as dye concentrations, solvents, incubation times, and temperatures. This study aimed to (1) systematically identify and optimize key factors that influence the staining of a wide range of polymers by analyzing four commercial textile dyes along with Nile red, one of the most common dyes used in MP analyses, and (2) develop a simple and versatile MP staining approach capable of effectively staining a range of environmentally relevant polymers with different morphologies.

## 2. Materials and Methods

### 2.1. Microplastics and Dye Selection

We used both virgin and weathered MPs in this study. Sources and key physical-chemical characteristics of these polymers are given in Table S1. Briefly, the pristine MPs were from the Hawaii Pacific University Polymer Kit 1.0, which was designed to “harmonize plastic pollution research”, and which included matching spectral data. However, because these MPs are relatively large, generally 1–5 mm pellets or beads, and because they are not fully representative of environmental MPs, we also produced MPs in our laboratory by cryogenically grinding weathered plastic debris collected from nearby Sardis Lake in north-central Mississippi. Plastics from the kit were used directly whereas the weathered plastic was washed and rinsed free of external debris prior to cryomilling. The cryomilled materials were typically 500–1000 µm in size. Together, our MPs represented 17 polymers commonly found in the environment. Each was stained with four different textile dyes (Table S2) along with Nile red dye for testing and comparison.

### 2.2. Microplastic Staining and Study Design

Several typical polymers (EPS, EVA, HDPE.1, LDPE-w, PA6-w, PEST, PP, and PVC.1) were selected to test the influence of dye concentration, staining temperature, and staining time (Table 1). Consistent with previous work [12], the ratio of MP weight (mg) to solution volume (mL) used was 5:1. Ultrapure water (purified, deionized, and 0.2 µm-filtered; Milli-Q; Millipore) was used to dilute textile dyes and Nile red since selected textile dyes are poorly soluble in methanol. Three replicates were performed for each treatment. A step-by-step procedure for staining MPs is given in Table S3.

To study the influence of dye concentration on staining, we prepared iDye dye concentrations at 100, 5, 2, 1, 0.5, 0.2 mg/mL in water, and Rit dye concentrations by diluting the concentrated dye solution 1:1, 1:20, 1:50, 1:100, 1:200, 1:500 in water, which corresponds to concentrations of 1100, 55, 22, 11, 5.5, 2.2 mg/mL, respectively. Nile red stock solution (1 mg/mL) was prepared with methanol and diluted to 10, 2, 0.5, 0.1, 0.02 µg/mL with DI water. The dyeing process occurred at 70 °C in 40 mL glass vials for 3 h in darkness. The stained MPs were then recovered by filtering through 45 µm mesh sieve. MPs were then suspended in methanol and DI water three times before being transferred to Petrislide dish, and dried at 30 °C.

To study the influence of incubation temperature on MP staining we tested select MPs at 4 °C, room temperature (21 °C), 40 °C, 70 °C and 100 °C for 3 h. The concentrations of iDye and Rit dyes were 5 and 55 mg/mL, respectively. Nile red solution was used as a control at 2 µg/mL.

To study the influence of incubation time on MP staining were incubated for 0.5, 1, 2, 3, and 5 h to examine the influence of dyeing time. The iDye dyes, Rit dyes, and Nile red were diluted to 5 mg/mL, 55 mg/mL, and 2 µg/mL, respectively, and MPs were stained at 70 °C.

To assess the dye stabilities, stained MPs were kept in the lake water, seawater, sediment slurry, and return activated sludge collected from University of Mississippi wastewater treatment plant for 1, 3, 5, and 7 days at room temperature under simulated sunlight (G2V Pico LED). Sediment solution was prepared at a concentration of 1 g/mL by mixing sediment and DI water. To better represent environmental samples, none of these solutions were sterilized.

The influence of solvent was tested by using the appropriate solvent (either methanol or water) to dilute 1 mg/mL Nile red stock solution to 2 µg/mL in methanol. For both solvents, 17 polymers were stained at 70 °C for 3 h and analyzed under identical conditions.

### 2.3. Characteristics of Stained Microplastics

Images of stained MPs were taken by a stereomicroscope (SteREO Discovery V12; Carl Zeiss Jena GmbH, Germany) equipped with 2 channel fluorescence (GFP and Cy3/Rhod/RFP) and an X-Cite 120Q fluorescence lamp illuminators. Large MPs (>500 µm) were imaged at 8× magnification and small MPs (<500 µm) were imaged at 40× magnification. This magnification approach is typically applied in visual enumeration of MPs in environmental samples [27,28,29]. The following two fluorescence ranges were chosen: green (excitation at 470/47 nm, emission at 525/50 nm) and red (excitation at 545/25 nm, emission at 605/70 nm). ImageJ image analysis software was used to assess fluorescence intensities as well as surface area of the stained MPs. Attenuated Total Reflection-Fourier Transform Infrared spectroscopy (ATR-FTIR, Cary 600, Agilent Technologies, Santa Clara, CA, USA) was utilized to collect IR spectra of MPs before and after dyeing.

## 3. Results and Discussion

### 3.1. Influence of Dye Concentration

**Figure 1:**
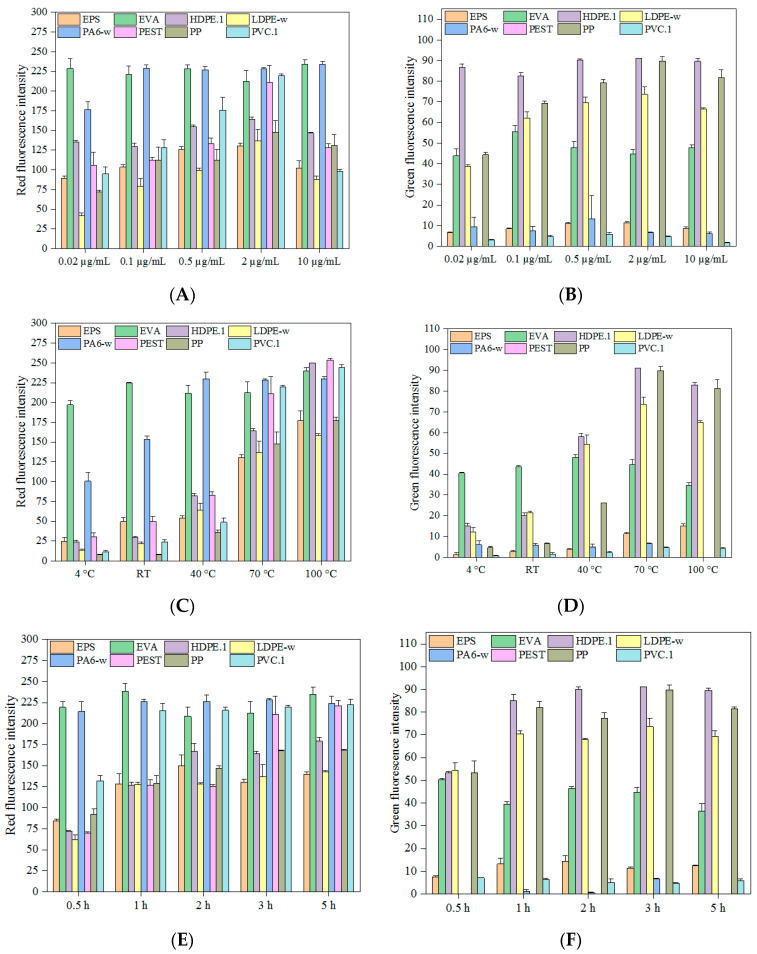
The influence of (**A**,**B**) concentrations of Nile red at 70 °C for 3 h, (**C**,**D**) temperatures at the Nile red concentration of 2 μg/mL for 3 h, and (**E**,**F**) time at the Nile red concentration of 2 μg/mL at 70 °C on microplastic staining.

The concentration of Nile red utilized for MP staining ranges from 0.1 µg/mL to more than 1 mg/mL in published literature [30,31,32,33,34,35]. In this study, we measured the green and red fluorescence intensities of polymers dyed with Nile red in concentrations ranging from 0.02 to 10 µg/mL. We found that all selected polymers exhibit visible red fluorescence across the tested concentrations. EVA showed the greatest affinity to Nile red, as evidenced by its strong yellow-gold color (even at 0.02 µg/mL). Red fluorescence intensity increased when the concentration was raised from 0.02 to 2 µg/mL (Figure 1). Beyond this point, however, most polymers showed a decrease in red fluorescence intensity as the Nile red concentration rose to 10 µg/mL. These findings are similar to other research, which found that the ideal concentration of Nile red lies between 0.1 and 2 µg/mL [31]. PE stained with Nile red at concentrations from 0.005 to 5 µg/mL, with slight quenching at 5 µg/mL, resulted in an average increase of the fluorescence intensity of PE by a factor of 3.1 [26]. Due to intermolecular interactions at higher concentrations, Nile red aggregates, and hence, a decrease in fluorescence intensity known as quenching occurs [36]. We also found that green fluorescence intensities of tested polymers were significantly weaker than red ones (Independent-samples *t*-test, *p* < 0.001). Non-polar polymers (LDPE-w, HDPE.1 PP, and to a lesser extent, EPS) showed relatively strong green fluorescence (Figure 1) compared to polar polymers, and PEST fibers did not exhibit visible green fluorescence at any tested Nile red concentrations. As with the trend of red fluorescence intensity, the strongest green fluorescence intensity was also found at a Nile red concentration of 2 µg/mL. We therefore used a Nile red concentration of 2 µg/mL for all further experiments.

Pink textile dyes showed a better staining effect than blue dyes (*p* < 0.001). Several MPs stained by textile dyes (EPS, HDPE.1, LDPE-w, PP) showed a similar trend with Nile red with the increase in dye concentrations (Figure S2). However, the highest concentration of textile dyes used here blocked the fluorescence intensities of stained polymers. Other polymers exhibited relatively constant red and green fluorescence intensities across dye concentrations. Red fluorescence intensities of tested polymers were significantly stronger than green ones (*p* < 0.001) with the majority of polymers barely observable under blue incident light. To obtain the strongest MP fluorescence intensities, we selected standard concentrations of 55 mg/mL for Rit dyes and 5 mg/mL for iDye dyes for further MP staining experiments.

All the tested dyes were friendly to polymers since we did not observe any surface area changes or mass loss in the samples. Because polymers were universally red fluorescent and had stronger red than green fluorescence signals, we recommend using the red fluorescence range for the analysis of stained MPs.

### 3.2. Influence of Incubation Temperature

**Figure 2:**
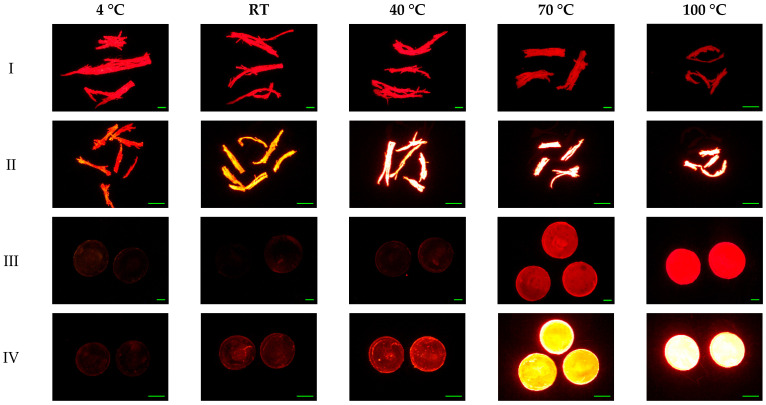
Red fluorescence microscope images of (**I**) PA6-w fragments stained by 55 mg/mL Rit pink dye, (**II**) PA6-w stained by 2 µg/mL Nile red, (**III**) PVC.1 stained by 5 mg/mL iDye pink dye, and (**IV**) PVC.1 stained by 2 µg/mL Nile red at different temperatures for 3 h. Scale bar denotes 1 mm.

Since the majority of MPs have melting points higher than 110 °C, the highest temperature tested here was 100 °C. MPs were observed to show stronger fluorescent signals with the increase in temperature. The strongest fluorescence intensities were present at 100 °C with the exception of PA6-w particles. Other researchers observed that at room temperature dyes were adsorbed on the surface of the polymer, and suggested that heating could facilitate the penetration of dyes into MPs [36]. This may occur due to increasing temperature loosening the macromolecular polymer chain as well as allowing the dye molecules to acquire sufficient energy to overcome intermolecular forces at adsorption sites [37]. When the temperature is then reduced to room temperature, the loose structure condenses, and the dye molecules become encapsulated in the MPs. The fact that some of the MPs, such as EPS, PVC.1, PEST, and PA6-w, changed from rigid glassy plastics to rubbery polymers with increased temperature provides evidence for this mechanism of action. In this rubbery state, a large amount of free volume between molecules is present [12,38]. This allowed the dye to be encapsulated rather than just adsorbed. As a result, fluorescence intensities were significantly higher at temperatures above the polymer’s glass transition temperatures than those below it (*p* = 0.007; Figure 2). The transition temperature of PA6-w is 60 °C, and stronger fluorescence intensity was expected to be found with staining temperatures higher than 60 °C. However, PA6-w fragments stained by textile dye showed lower red fluorescence intensity while the staining temperature was above 70 °C (Figure S3). Other polymers (EVA, LDPE-w, HDPE.1, and PP) are in rubbery states at room temperature, and increased temperature significantly enhanced the fluorescence intensities, although a decrease was observed on textile dye-stained EVA when the temperature exceeded 70 °C. The soft and flexible structure of EVA fragments may facilitate the adsorption of dyes. However, all low transition temperature polymers exhibited a rapid increase in fluorescence intensity with increasing temperature.

Raising the temperature did not substantially increase the fluorescence intensities of MPs stained with blue dyes. MPs stained by both dyes showed relatively low fluorescence intensities across all incubation temperatures, suggesting that blue textile dyes are not suitable for fluorescent staining. Morphological changes were not observed among selected polymers, except for EVA and PA6-w fragments, which were observed to be slightly deformed at 100 °C (Figure 2).

### 3.3. Influence of Staining Time

A literature search of incubation times for MP staining found that they ranged from 5 min to more than 24 h, with a majority of studies adopting 30 min [2,37,39]. In this study, all tested polymers stained by Nile red showed strong red fluorescence intensities after 30 min of staining, especially EVA and PA6-w fragments, which rapidly reached their strongest red fluorescence intensities within 0.5 h (Figure 1). Other polymers (e.g., HDPE.1, PP, and PEST) were fully stained by Nile red within 3 h. These results are consistent with observations of other researchers that PE, PS, and PET stained by Nile red showed increased red fluorescence intensity with the extension of staining time [36]. Our results also showed that green fluorescence intensities of Nile Red stained polymers demonstrated a similar trend, except for several non-green fluorescent polymers (PA6-w and PEST).

Similar to Nile red, textile dyes showed good affinity to EVA and PA6-w fragments, giving strong fluorescent signals within 30 min. However, the fluorescent signals of PA decreased with the increase in staining time. In contrast, the signals for non-polar polymers (EPS, LDPE-w, HDPE.1, and PP) were initially weak but substantially increased with the extension of staining time. Maximal fluorescent signals were achieved in 3 h for polymers stained with textile, even though their maximum fluorescent signals were relatively weaker compared to Nile red.

We did not find any significant mass variation or surface area deviation among the tested MPs after 5-h staining. Given this lack of damage, combined with the time needed for textile dyes to reach maximum intensity, we utilized a standard 3 h incubation time for subsequent studies.

### 3.4. Staining Efficiency on Various Polymers

To assess the staining efficacy of Nile Red and pink textile dyes, both transparent and colored, as well as weathered and virgin MPs were used. Nile red-stained MPs can fluoresce when irradiated under UV, blue, green, and red incident lights with excitation wavelengths varying from 254 to 580 nm [23,32,40]. Blue fluorescence is typically avoided as certain polymers (e.g., LDPE, PP, EPS) have no fluorescent response to blue excitation. Green-yellow fluorescence (excitation at 470/40 nm, emission at 540/50 nm) has been suggested as the best choice for MP detection as it can sometimes distinguish natural particles and plastics [23,26]. However, this may also not be suitable for all polymers. Studies have found that PEST, PET, and PA stained by 5 µg/mL Nile red had a barely detectable, dim glow of green fluorescence and that polyurethane, PC, PVC, and PET dyed by 1 µg/mL Nile red exhibited weak green fluorescence [23,41]. We also found very weak green fluorescent signals for Nile Red stained PC, PA-w, and PETE-w. While biological tissues can also exhibit strong red fluorescence, most MP extraction methods focus on the removal of such tissues [41]. Due to this, and the fact that all polymers exhibited higher intensities of red fluorescence than green, we chose to focus our investigation of polymer staining efficiency on their red fluorescence signals. Usable levels of red fluorescence were observed in all polymers, even in the colored plastics. Previous studies have reported that the presence of additive dyes affects MPs affinity to staining dyes, which was consistent with our results [37,41,42]. We found that only the edges of black ABS stained by textile dyes showed red fluorescence (Figure S5). The Nile red-stained black ABS pellets showed a red intensity of 59.8 ± 2.8 and a green fluorescence intensity of 0, which increased to 221.4 ± 1.8 and 10.1 ± 0.1 in clear ABS pellets, respectively. Similarly, other researchers demonstrated that brown HDPE and black PP were stained only around their edges, and red PA, black polyester, and blue acrylic fibers were not stained by Nile red [42].

To assess our staining methods, commercially available fluorescent PE microbeads (300–330 µm) were used as references. The red and green fluorescence intensities of these microbeads were 255 and 8.4 ± 0.2 at 8× magnification, respectively. The average red and green fluorescence intensities for different forms of PE in this study were 193.8 ± 39.5 and 87.1 ± 6.8, respectively, which are comparable with those of the commercial PE microbeads.

Our results did not demonstrate a relationship between polymer hydrophobicity and its red fluorescence intensity. The more hydrophobic polymers such as PP, PS, and some forms of PE did not show stronger red fluorescence intensities than less hydrophobic polymers (e.g., PET, PVC, EVA, PA, ABS) (*p* = 0.275). However, weaker green fluorescent signals were observed for these less hydrophobic polymers (*p* < 0.001). This is similar to the literature, which reports that non-polar PE and PP particles had the strongest green fluorescence intensity, followed by the intermediate polar PEST and PA, and highly polar PVC only showed a very weak signal [26]. No clear correlation between the polarity of the polymers and their fluorescence intensity was found among textile dyes either. For example, polar polymers stained by Rit pink dye had higher red fluorescent signals but not significant (*p* = 0.601). For these samples, fluorescent intensity was more related to the color of the dye used, as both iDye pink dye and Rit pink dye resulted in stronger fluorescence intensities than the blue textile dyes (Figure S6). This is consistent with results from a previous study [12].

**Figure 3:**
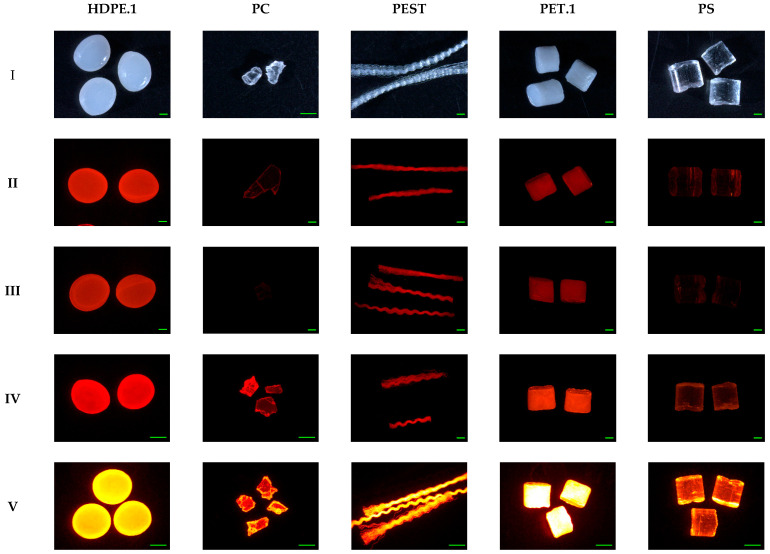
Fluorescence microscope images of (**I**) original color of polymers, (**II**) polymers stained by 55 mg/mL Rit pink dye, (**III**) polymers stained by 5 mg/mL iDye pink dye, (**IV**) polymers stained by 2 µg/mL methanol-based Nile red, and (**V**) polymers stained by 2 µg/mL water-based Nile red at 70 °C for 3 h. Scale bar denotes 1 mm.

**Figure 4:**
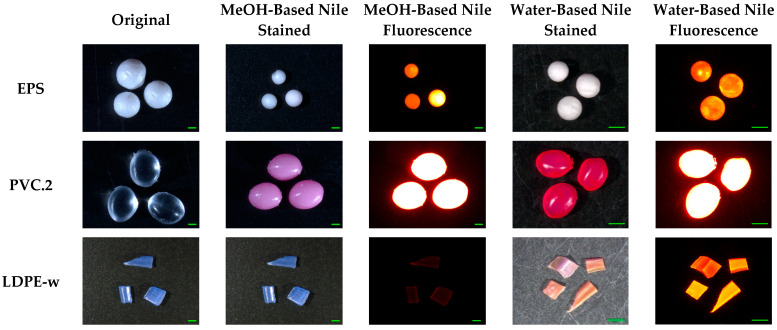
Fluorescence microscope images of EPS, PVC.2, and LDPE-w stained by 2 µg/mL methanol-based Nile red and 2 µg/mL water-based Nile red at 70 °C for 3 h. Scale bar denotes 1 mm.

Among all these tested polymers, Nile red-labeled plastics are more likely to be used for MP staining than textile dyes due to the weaker fluorescence of textile dyes. Polymers stained by pink dyes showed relatively stronger red fluorescent signals than blue textile dyes, and weak or no green fluorescent signals were widely found. EPS foams stained with Nile red and textile dyes did not show extremely strong fluorescence intensity, although they have mesoporous structures. However, Shim et al. (2016) observed that EPS foams were brightly stained with Nile red in n-hexane compared to spherical PE particles. Staining MPs with Nile red under different solvents influences MP fluorescence intensity, which may be associated with the solvatochromism of Nile red (Figure 3, Figure S6) [26,36,39]. As the increasing of solvent polarity, the solvatochromic effect markedly caused red shifts in the fluorescence emission spectrum, especially for non-polar polymers since, as Martinez and Henary noted, “solvents with large dipole moments are better able to stabilize the Franck-Condon excited state” and in the polar solvent (water), the lipophilic Nile red absorbs more strongly onto non-polar MPs than in the less polar organic solvent [23,43]. LDPE-w, PP, and PS pellets stained by methanol-based Nile red showed red fluorescence intensities of 12.8 ± 0.6, 22.9 ± 0.3, and 26.9 ± 0.9, respectively. These increased to 137.0 ± 13.9, 147.7 ± 14.7, and 108.2 ± 1.0 when the solvent was changed to water (Figure 4). Using methanol as a solvent may also have other disadvantages, as it may cause damage to certain types of plastics. EPS foam MPs shrunk by 25.1% and 61.9% when the temperature increased to 70 °C and 100 °C, respectively. This damage may be avoided by reducing the incubation temperature, as these MPs were intact when the temperature was not higher than 40 °C. However, the trade-off in using a lower incubation temperature is a decrease in the stained MPs fluorescence intensity. EPS was not the only polymer to experience changes when stained with a methanol-based solution. PVC.2, which contains phthalates as an additive, became rigid and tough after staining by methanol-based Nile red as well as losing 10.23% of its mass (Figure 4). This may be due to the leaching of phthalates from polymers into solvents. Meanwhile, methanol penetrated into the PVC.2 since methanol C-O vibration at 1020 cm^−1^ was observed in PVC.2 stained by the MeOH-based Nile red (Figure S7). Weak green fluorescent signals of PS, EPS, PEST, PA6, PA66, PETE-w, PVC.1, and PC stained by either water-based Nile red or methanol-based Nile red were found, but these polymers showed strong red fluorescent signals, which is consistent with other studies [32,36,41]. Weathered plastics can be more polar due to the formation of carbonyl groups through photo-oxidation and altered surface roughness [44]. However, we did not find a significant increase in the fluorescence intensities of weathered polymers. Therefore, it may be the MPs polymer type that is the dominant factor for staining.

In fluorescence microscopy, the magnification used to observe MPs affects the fluorescence intensity [42]. As such, we chose 8× and 40× as standard magnifications to observe all MPs. Our CA particles had a size of 390 ± 290 µm. Pink textile dyes and water-based Nile red resulted in the maximal red fluorescence intensity (255) at 40× magnifications. However, at 8× magnification, the intensities dropped to 13.4 ± 2.9 and 145.4 ± 15.9, respectively. This suggests that smaller sizes of MPs may appear to show stronger fluorescent signals, as they will be observed at higher magnifications.

Compared with undyed MPs, there was no significant change in the surface morphology after 3-h staining at 70 °C. Additionally, identical FTIR spectra were observed for all polymers before and after staining (Figure S7), which corroborates that these dyes are usually not covalently bonded to polymers, but, according to Gewert et al., adsorbed to them via “van der Waals interactions with additional dipole interactions” in polar polymer types [23,44].

### 3.5. Stability of Fluorescence

Fluorescence stability was assessed for the pink textile dyes along with Nile Red, due to their higher intensities. Stained particles were placed in four common solutions (freshwater, seawater, sediment slurry, and returned activated sludge) to test the fluorescence stability at room temperature. All these dyes were fluorescently stable under these solutions for 7 days under simulated sunlight (Figure S8). This stability is attributed to strong interactions between polymers and dyes due to their adsorption to the surface or incorporation into polymers. While these results are promising, more study is needed on their stabilities over longer times and in harsher solutions. Red fluorescence intensities of MPs stained by Nile red at room temperature were observed to reduce significantly after one month due to the easy desorption of dye from the surface of polymers [36]. KOH and non-polar solutions, which are common in chemical digestions for MP extractions, have also been reported to weaken the fluorescence intensities of LDPE fragments, HDPE microbeads, and polyacrylonitrile fibers stained at 70 °C [12].

## 4. Conclusions

Dye concentration, incubation time, and temperature greatly affect the fluorescence intensity of stained polymers, with red fluorescent signals being stronger than green ones. Commercially available textile dyes and Nile red are effectively adsorbed to miscellaneous polymers under tested conditions. However, the low fluorescence intensities of blue textile dyes make them unsuitable for MP staining. As demonstrated in this study, Nile red has advantages over textile dyes, such as its increased fluorescence intensity and good affinity for a wide range of polymers, but pink textile dyes also have applicable potential. The staining procedure did not significantly alter the surface, mass, and chemical characteristics of the particles, based on FTIR and stereomicroscopy. The stability of these textile dyes in the four solutions meant to mimic environmental matrices also speaks to their suitability for spiking applications. While Nile Red still remains the optimal choice for MP staining, pink textile dyes represent a cheaper, yet still potentially viable option.

## Figures and Tables

**Table 1:** The design of staining experiments.

Influencing Factors	Dyes	Series	Polymers
Dye concentration ^(1)^	Nile red	0.02, 0.1, 0.5, 2, 10 (µg/mL)	EPS, EVA, HDPE.1, LDPE-w, PA6-w, PEST, PP, PVC.1
	Rit pink dye	1:500, 1:200, 1:100, 1:50, 1:20, 1:1 or 2.2, 5.5, 11, 22, 55, 1100 (mg/mL)	
	Rit blue dye		
	iDye pink dye	0.2, 0.5, 1, 2, 5, 100 (mg/mL)	
	iDye Blue dye		
Incubation temperature ^(2)^	Nile red	4° C, RT (21 °C), 40 °C, 70 °C and 100 °C for 3 h	
	Rit pink dye		
	Rit blue dye		
	iDye pink dye		
	iDye blue dye		
Incubation duration ^(2)^	Nile red	0.5, 1, 2, 3, 5 h at 70°C	
	Rit pink dye		
	Rit blue dye		
	iDye pink dye		
	iDye blue dye		

^(1)^ Performed at 70 °C and 3 h; ^(2)^ concentrations at 5 mg/mL (iDye dyes), 55 mg/mL (Rit dyes), and 2 µg/mL (Nile red).

## Data Availability

Data is available upon request.

## Supplementary Materials

The following supporting information can be downloaded at: https://www.mdpi.com/article/10.3390/molecules27217415/s1, Figure S1: Green fluorescence microscopic images of polymers stained by 2 µg/mL Nile red at 70 °C for 3 h. Scale bar denotes 1 mm; Figure S2: The influence of dye concentrations on microplastic staining at 70 °C for 3 h. (A) Rit pink dye; (B) Rit blue dye; (C) iDye pink dye; (D) iDye blue dye; Figure S3: The influence of temperatures on microplastic staining with Rit dyes and iDye dyes concentrations of 55 mg/mL and 5 mg/mL for 3 h. (A) Rit pink dye; (B) Rit blue dye; (C) iDye pink dye; (D) iDye blue dye; Figure S4: The influence of time on microplastic staining with Rit dyes and iDye dyes concentrations of 55 mg/mL and 5 mg/mL at 70 °C. (A): Rit pink dye; (B) Rit blue dye; (C) iDye pink dye; D: iDye blue dye; Figure S5: Red fluorescence microscope images of black ABS pellets stained by (A) 55 mg/mL Rit pink dye, (B) 5 mg/mL iDye pink dye, and (C) 2 mg/mL Nile red, and clear ABS pellets stained by (D) 2 mg/mL Nile red at 70 °C for 3 h. Scale bars denote 1000 µm; Figure S6: Different polymers stained by textile dyes (A,B,E,F) and Nile red (C,D,G,H) with variable red fluorescence intensities; Figure S7: FTIR spectrum of LDPE.1, PA6, PET.1, PVC.2, and PS stained by different dyes; Figure S8: Fluorescence intensity stability of Rit pink dye, iDye pink dye, and water-based Nile red for 7 days in lake water, seawater, sediment slurry, and activated sludge slurry; Table S1: Summary of polymers’ characteristics and sources; Table S2: Information of applied dyes; Table S3: Step-by-step procedure for staining of microplastics with fluorescent dyes.

## Sample Availability

Not available.

## Nomenclature

Ultra-low-density ethylene	ULDPE
Low-density polyethylene	LDPE
Linear low-density polyethylene	LLDPE
Medium-density polyethylene	MDPE
High-density polyethylene	HDPE
Polypropylene	PP
Polyester	PEST
Polyethylene terephthalate	PET
Ethylene-vinyl acetate	EVA
Acrylonitrile-butadiene-styrene	ABS
Expanded polystyrene foam	EPS
Polystyrene	PS
Nylon 6	PA6
Nylon 6,6	PA66
Polyvinyl chloride	PVC
Cellulose acetate	CA
Weathered polycarbonate	PC
Weathered low-density polyethylene	LDPE-w
Weathered polyethylene terephthalate	PETE-w
Weathered nylon 6	PA6-w
