# Supplementary material for: Labeling Microplastics with Fluorescent Dyes for Detection, Recovery, and Degradation Experiments
Source: Molecules. 2022 Nov 1;27(21):7415. doi: 10.3390/molecules27217415 (PMC9653731; doi:10.3390/molecules27217415)
Supplement: Supplementary file 1 [file molecules-27-07415-s001.zip › molecules-1975149-supplementary.pdf]

Supplemental information

Labeling Microplastics with Fluorescent Dyes for Detection, Recovery, and Degradation Experiments

Zhiqiang Gao [1], Kendall Wontor [1] and James V. Cizdziel [1]*

[1] Department of Chemistry and Biochemistry, University of Mississippi, University, MS 38677, USA; zgao2@go.olemiss.edu (Z.G.); kwontor@go.olemiss.edu (K.W.)

* Correspondence:

Email address: cizdziel@olemiss.edu (J.V.C)

The Number of Pages: 9

The Number of Tables: 2

The Number of Figures: 8

Table S1. Summary of polymers' characteristics and sources.

Polymer	Abbreviation	Shape/Color	Size range (mm)*	Melting point (°C)	Glass transition (°C)
Ultra low-density ethylene	ULDPE	Pellet/Clear	5.1 ± 0.1	123.4	-110
Low-density polyethylene	LDPE.1	Pellet/Clear	4.5 ± 0.3	113.5	-110
Low-density polyethylene	LDPE.2	Pellet/Clear	4.7 ± 0.3	105.2	-110
Linear low-density polyethylene	LLDPE.1	Pellet/Clear	5.0 ± 0.1	122.4	-110
Linear low-density polyethylene	LLDPE.2	Pellet/White	5.1 ± 0.1	120.8	-110
Medium-density polyethylene	MDPE	Pellet/White	4.8 ± 0.2	124.4	-110
High-density polyethylene	HDPE.1	Pellet/White	4.8 ± 0.1	130.6	-110
High-density polyethylene	HDPE.2	Pellet/White	4.6 ± 0.2	134.8	-110
Polypropylene	PP	Pellet/Clear	4.2 ± 0.3	164.7	-10
Polyester	PEST	Fiber/White	10.0 ± 2.0	251.3	68.3
Polyethylene terephthalate	PET.1	Pellet/White	3.3 ± 0.4	243.5	80.2
Recycled polyethylene terephthalate	PET.2	Pellet/Yellowish	4.9 ± 0.2	254.7	73-78
Ethylene-vinyl acetate	EVA	Fragment/Clear	4.9 ± 0.4	87.2	-20
Acrylonitrile-butadiene-styrene	ABS	Pellet/Black	3.2 ± 0.1	~200	108.6
Expanded polystyrene foam	EPS	Foam/White	2.6 ± 0.8	270	106.5
Polystyrene	PS	Pellet/Clear	3.5 ± 0.2	270	103.4
Nylon 6	PA6	Pellet/White	3.3 ± 0.1	220.7	60
Nylon 6,6	PA66	Pellet/White	2.8 ± 0.2	259.9	55-58
Polyvinyl chloride	PVC.1	Pellet/Clear	4.2 ± 0.2	100-260	65-85
Polyvinyl chloride with phthalates	PVC.2	Pellet/Clear	4.1 ± 0.3	100-260	65-85
Cellulose acetate	CA	Fragment/White	0.39 ± 0.29	230-300	100-130
Weathered polycarbonate	PC	Fragment/Clear	0.5-1	230-260	140-150
Weathered low-density polyethylene	LDPE-w	Fragment/Blue	1-3	85-125	-110
Weathered polyethylene terephthalate	PETE-w	Film/Clear	1-3	245-265	73-78
Weathered nylon 6	PA6-w	Fragment/Yellowish	0.5-1	210-220	60

*Major dimensions were measured for irregular MPs and diameters were reported for spherical MPs. Melting point and glass transition temperatures were either provided by Hawaii Pacific University, PerkinElmer, and Omnexus. LLDPE.1: ethylene-octene copolymer; LLDPE.2: LLDPE made with metallocene catalyst; HDPE.1: ethylene-hexene copolymer; HDPE.2: regular high-density polyethylene.

Table S2. Information of applied dyes.

Dye (product number)	State	Density (g/mL)	Supplier
iDye blue (JID 451)	Solid	/	Jacquard
iDye pink (JID 456)	Solid	/	Jacquard
Rit pink (2095)	Liquid	1.086	Rit
Rit blue (2283)	Liquid	1.072	Rit
Nile red (N3013)	Solid	/	Sigma-Aldrich

Table S3. Step-by-step procedure for staining of microplastics with fluorescent dyes.

Step	Action
1	Weigh out the MPs for staining and record its mass.
2	Based on the type of dye, dilute or dissolve the dye to the following concentrations using deionized (DI) water (Rit dye: 55 mg/mL; iDye dye: 5 mg/mL; Nile red: 2 μg/mL).
3	Mix MPs with the prepared dye in a ratio of 5 mg of MP:1 mL of dye in a clean glass vial.
4	Heat the mixture at 70°C for 3 hours. Use an oven mat or rack to avoid direct contact between the vial and the oven bottom. Directly placing the vials onto the oven floor may result in damage to any MPs on the bottom of the vial due to the high temperature.
5	After staining, thoroughly rinse the MPs with methanol and DI water to remove any dye residues attached to the surface.
6	Dry the rinsed MPs in a laminar fume hood. Store at room temperature until use.

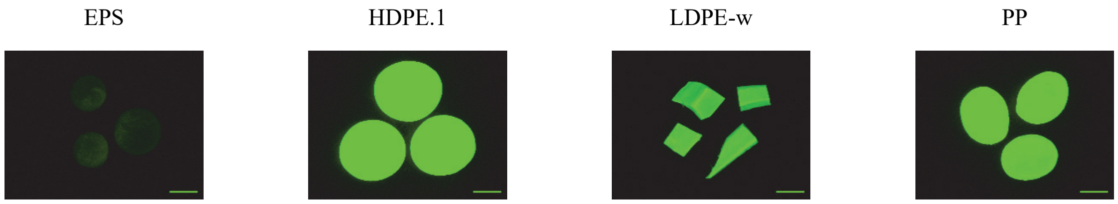

Figure S1. Green fluorescence microscopic images of polymers stained by 2 μg/mL Nile red at 70°C for 3 hours. Scale bar denotes 1 mm.

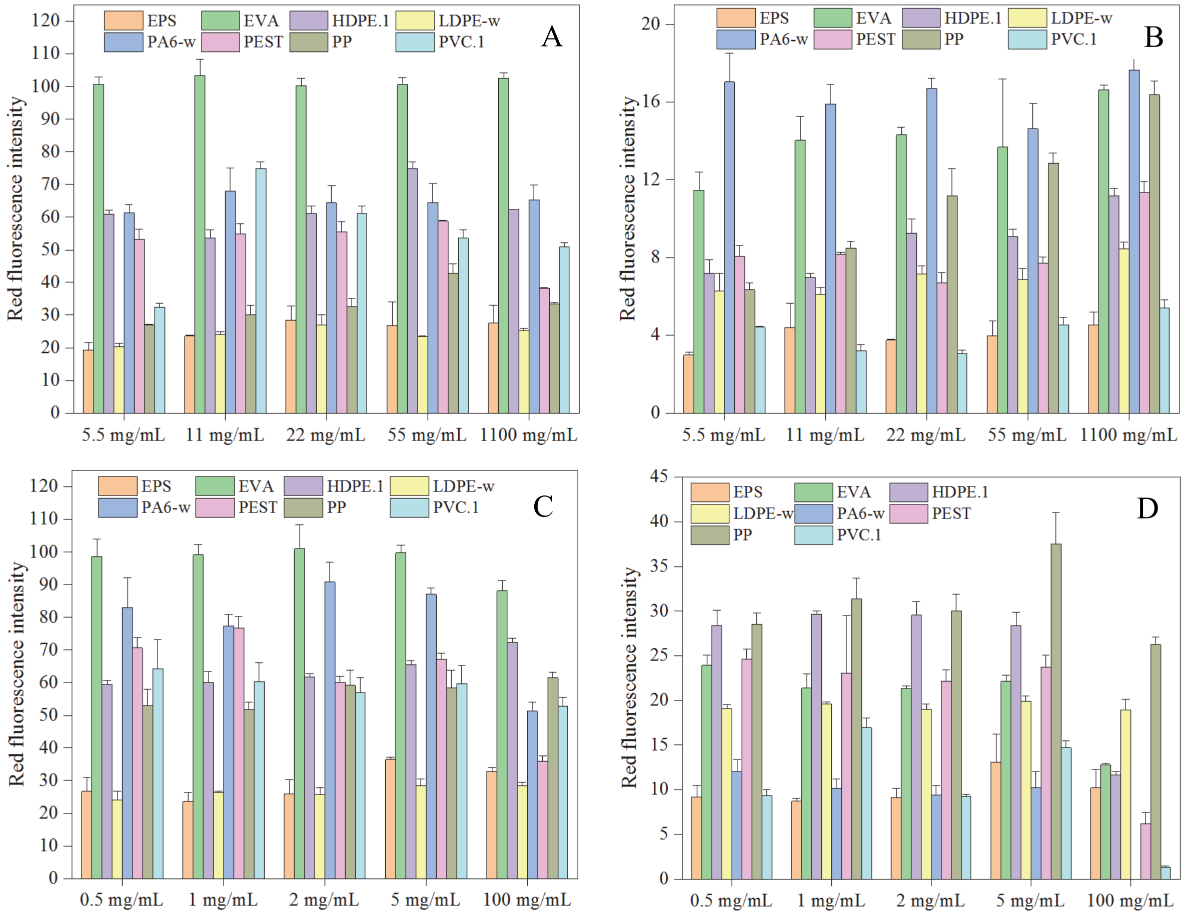

Figure S2. The influence of dye concentrations on microplastic staining at 70 °C for 3 h. A: Rit pink dye; B: Rit blue dye; C: iDye pink dye; D: iDye blue dye.

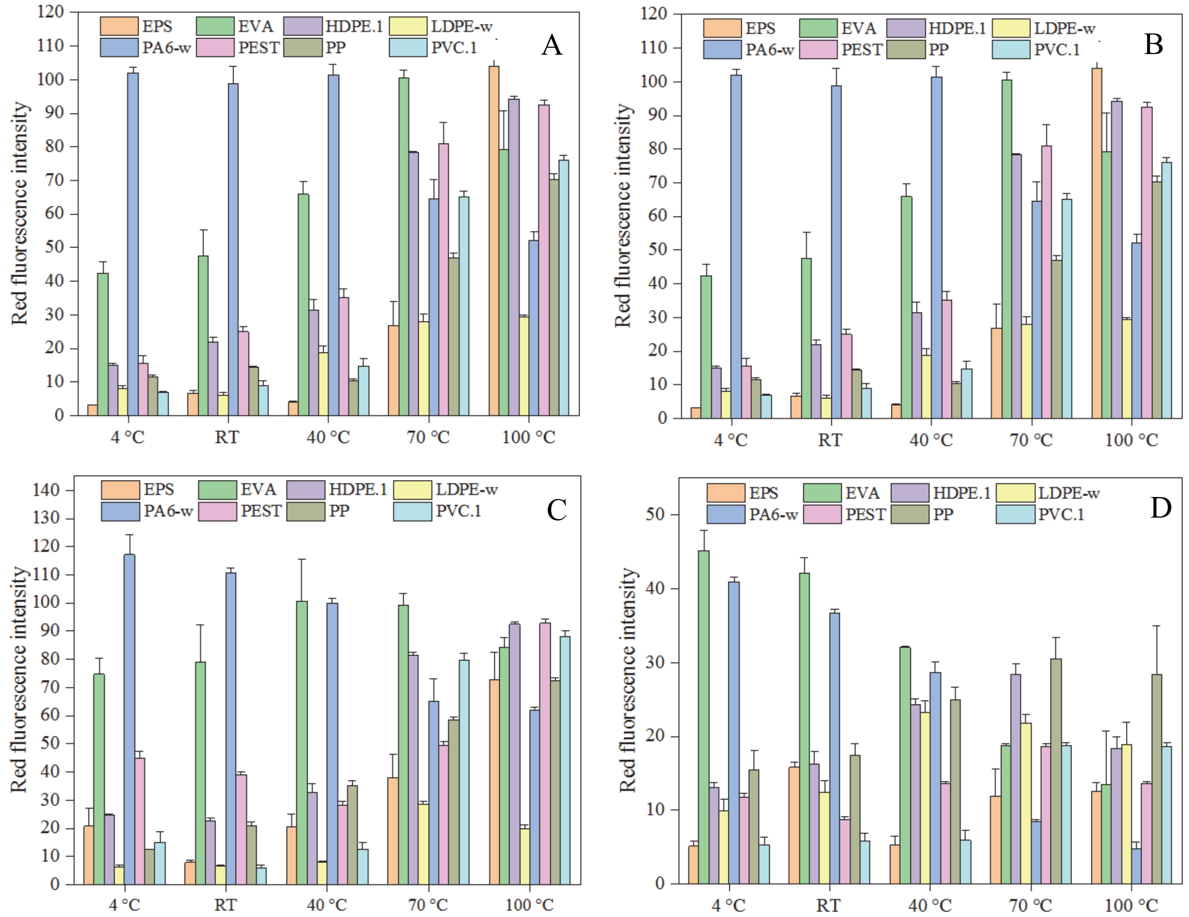

Figure S3. The influence of temperatures on microplastic staining with Rit dyes and iDye dyes concentrations of 55 mg/mL and 5 mg/mL for 3 h. A: Rit pink dye; B: Rit blue dye; C: iDye pink dye; D: iDye blue dye.

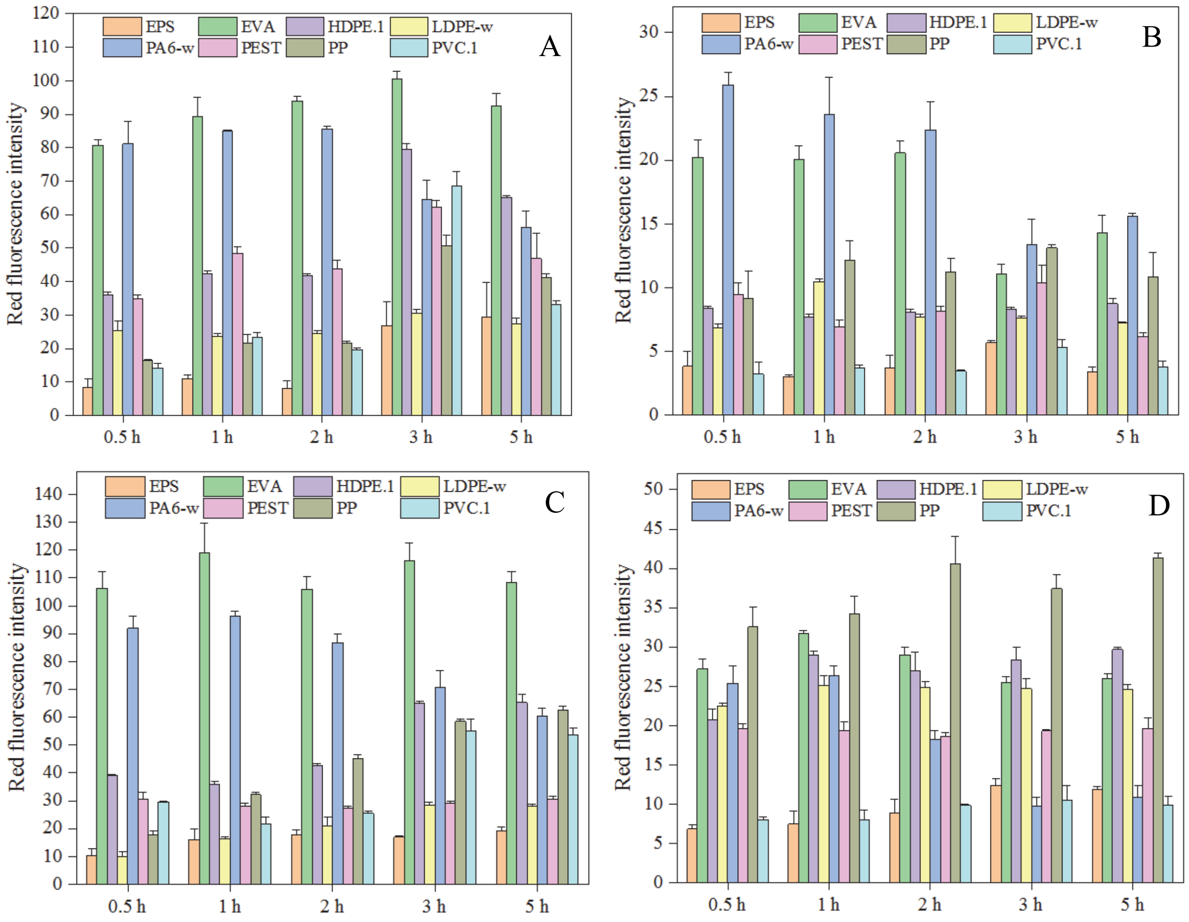

Figure S4. The influence of time on microplastic staining with Rit dyes and iDye dyes concentrations of 55 mg/mL and 5 mg/mL at 70 °C. A: Rit pink dye; B: Rit blue dye; C: iDye pink dye; D: iDye blue dye.

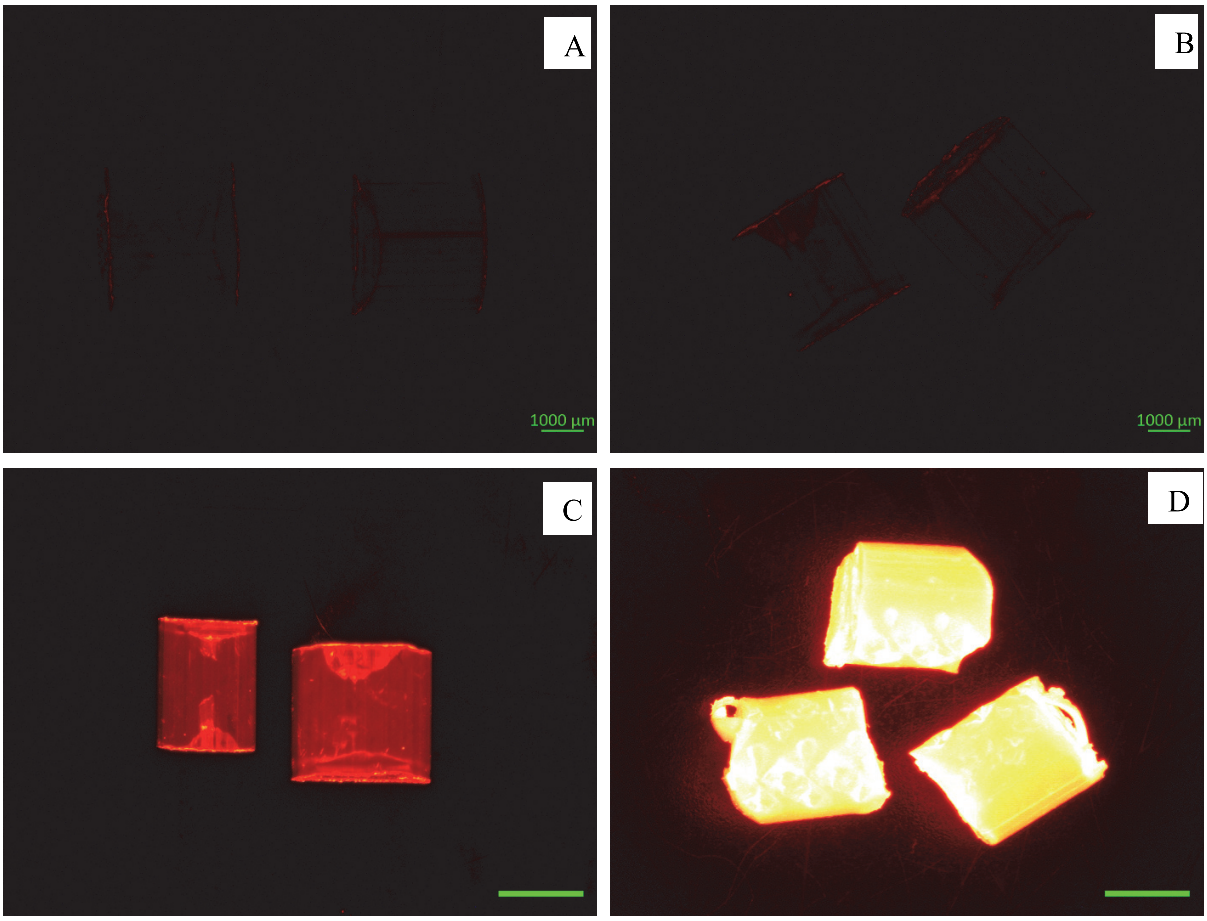

Figure S5. Red fluorescence microscope images of black ABS pellets stained by (A) 55 mg/mL Rit pink dye, (B) 5 mg/mL iDye pink dye, and (C) 2 mg/mL Nile red, and clear ABS pellets stained by (D) 2 mg/mL Nile red at 70 °C for 3 hours. Scale bars denote 1000 µm.

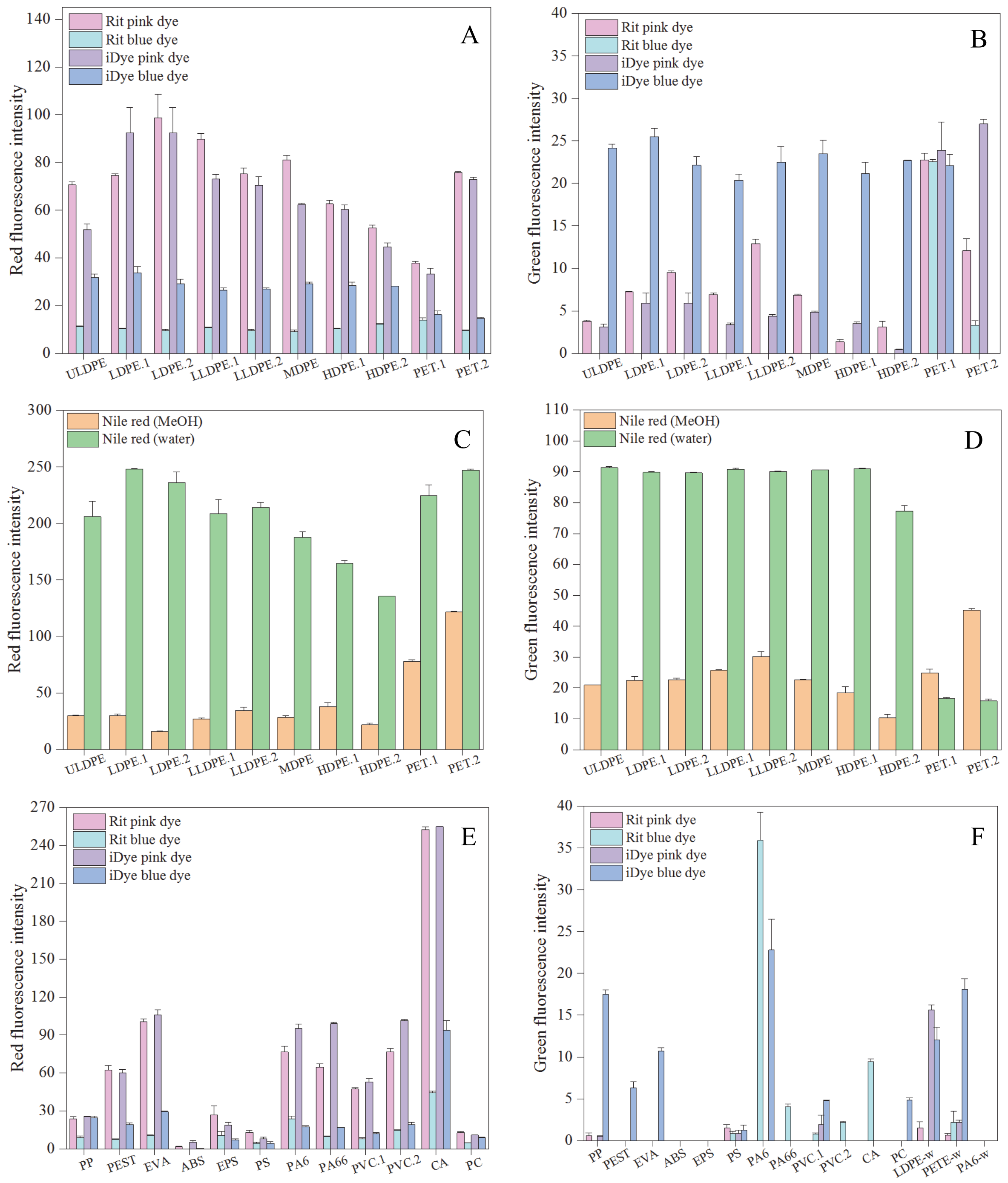

A
B
C
D
E
F
Rit pink dye
Rit blue dye
iDye pink dye
iDye blue dye
Nile red (MeOH)
Nile red (water)
Red fluorescence intensity
Green fluorescence intensity
ULDPE
LDPE.1
LDPE.2
LLDPE.1
LLDPE.2
MDPE
HDPE.1
HDPE.2
PET.1
PET.2
PP
PEST
EVA
ABS
EPS
PS
PA6
PA66
PVC.1
PVC.2
CA
PC
LDPE-w
PETE-w
PA6-w

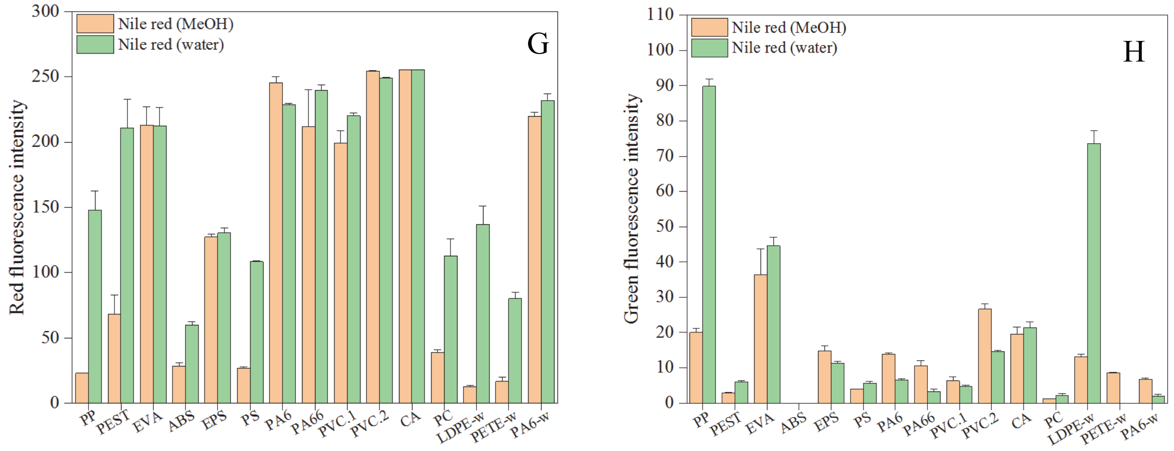

Figure S6. Different polymers stained by textile dyes (A, B, E and F) and Nile red (C, D, G and H) with variable red fluorescence intensities.

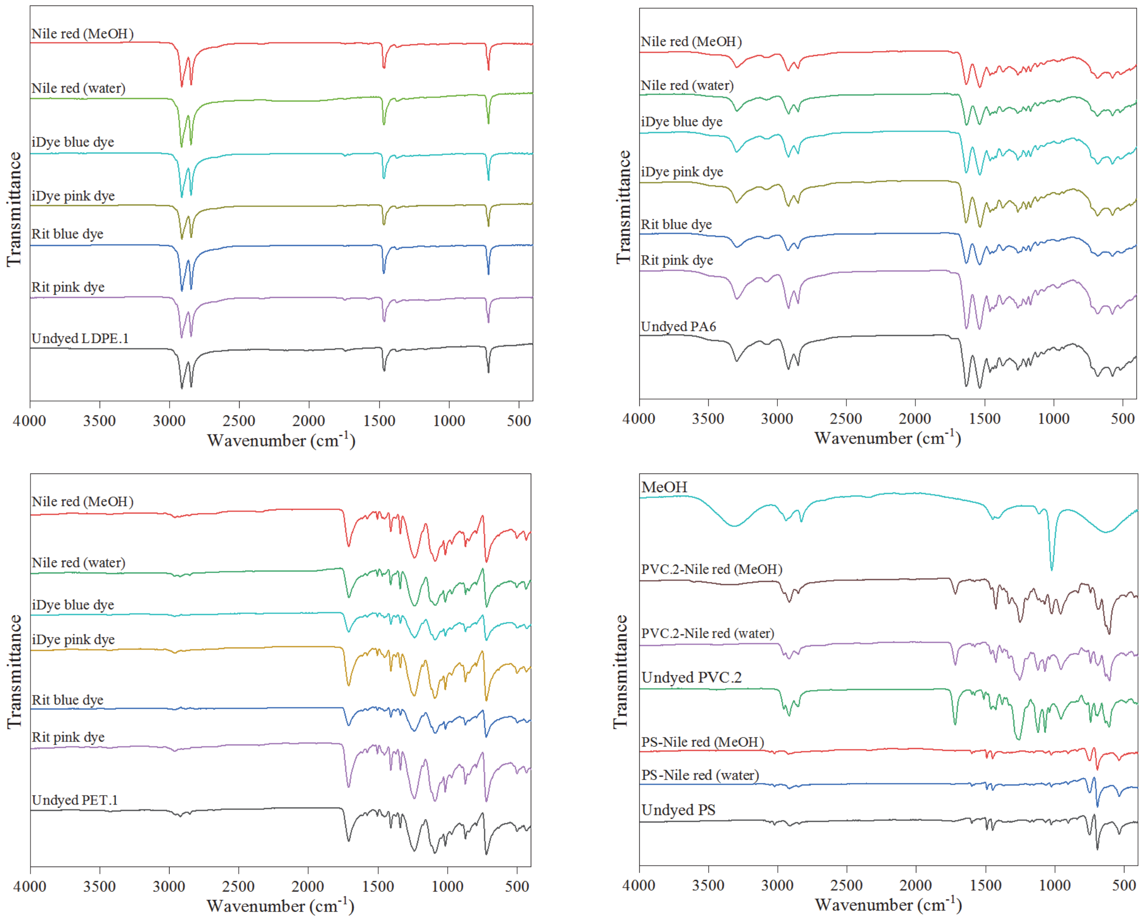

Figure S7. FTIR spectrum of LDPE.1, PA6, PET.1, PVC.2, and PS stained by different dyes.

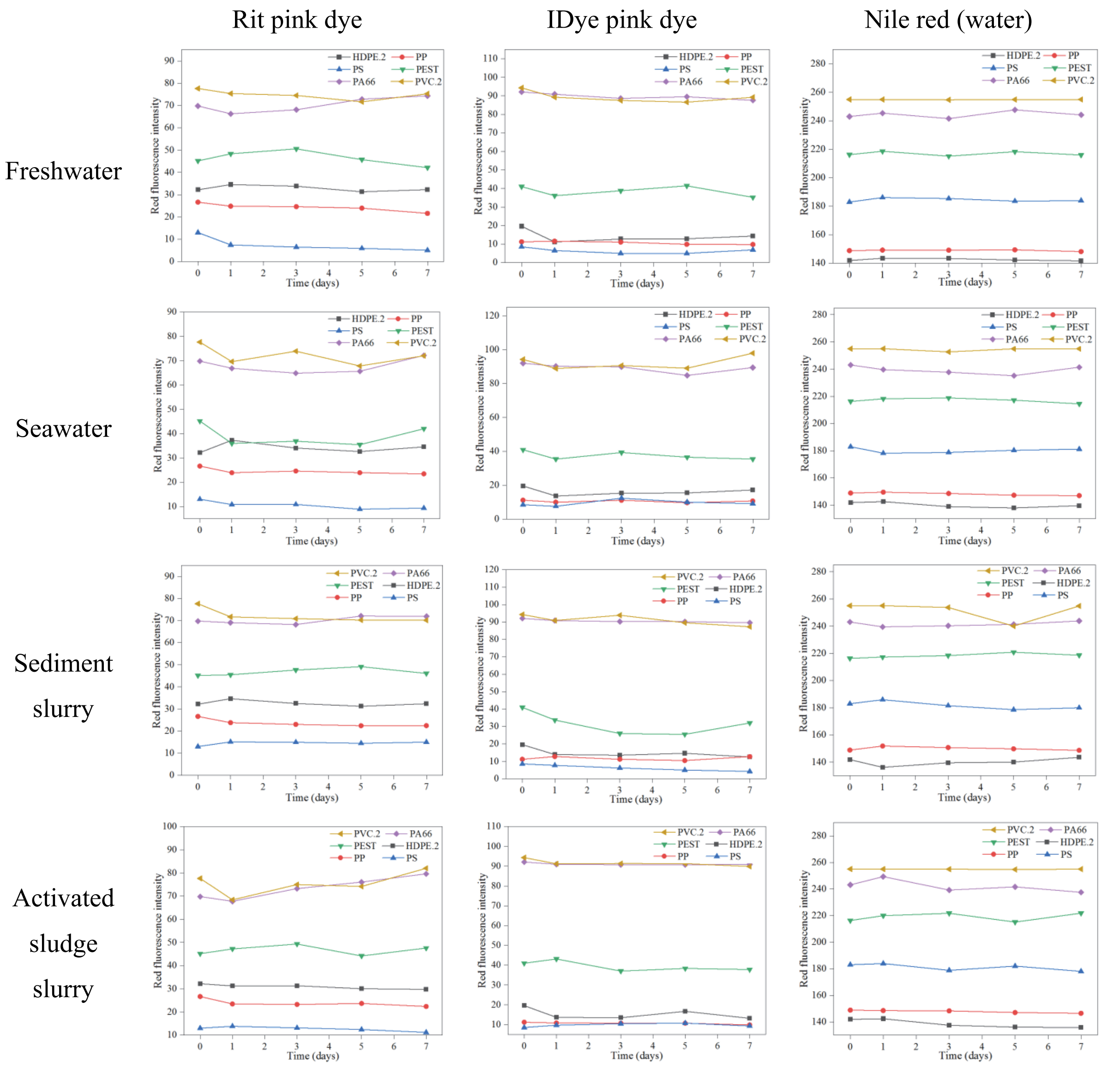

Figure S8. Fluorescence intensity stability of Rit pink dye, iDye pink dye and water-based Nile red for 7 days in lake water, seawater, sediment slurry and activated sludge slurry.
